# Nitric oxide inhalation as an interventional rescue therapy for COVID-19-induced acute respiratory distress syndrome

**DOI:** 10.1186/s13613-020-00681-9

**Published:** 2020-05-20

**Authors:** Jun Kobayashi, Isamu Murata

**Affiliations:** Faculty of Pharmacy & Pharmaceutical Science, Josai University, 1-1 Keyakidai, Sakado, Saitama, 350-0295 Japan

## Abstract

COVID-19 is an emerging disease of public health concern. While there is no specific recommended treatment for COVID-19, nitric oxide has the potential to be of therapeutic value for managing acute respiratory distress syndrome in patients with COVID-19. However, inhaled nitric oxide has not yet been formally evaluated. Given the extent of the COVID-19 pandemic, and the large numbers of hospitalized patients requiring respiratory support, clinical use of inhaled nitric oxide may become an alternate rescue therapy before extracorporeal membrane oxygenation for the management of acute respiratory distress syndrome in patients with COVID-19.

Novel coronavirus disease 2019 (COVID-19) is an emerging disease of public health concern, and the current pandemic is having a major global impact. Increasing attention is being focused on the development of therapeutic strategies against this disease. We read, with great interest, the article by Li et al. “Therapeutic strategies for critically ill patients with COVID-19” published in this journal [1]. While there is no specific recommended antiviral treatment, and vaccines have yet to be developed, the authors provided a powerful pharmacological strategy for the treatment of critically ill patients with COVID-19 acute respiratory distress syndrome (ARDS). In this review article, the drug applications for COVID-19 are well described according to disease severity; however, nitric oxide (NO) inhalation therapy, which is not described in this review, may be included in the strategy as a promising therapeutic candidate. In 2004, during the severe acute respiratory syndrome coronavirus (SARS-CoV) outbreak, a pilot study showed that low-dose inhaled NO (max 30 ppm) could shorten the time of ventilatory support for patients infected with SARS-CoV [2]. Although epidemiological evidence supporting the use of inhaled NO in treating COVID-19 has not yet been identified, similar therapeutic effects of NO can be expected for patients with COVID-19 due to the genetic similarities between the two viruses [3]. Based on this experience, clinical trials have begun in several medical institutes in the United States, and now a phase 2 clinical trial of inhaled NO is being conducted for mechanically ventilated patients with COVID‑19 ARDS to confirm whether NO inhalation will become an interventional therapy to rescue patients with this disease [4].

The inflammatory cytokine storm induced by virus infection is closely related to the development and progression of ARDS. Given the common pathological process leading to virus-induced ARDS [3], previous experience suggests that inhaled NO may be useful for managing COVID-19 ARDS. Previously published in vitro studies indicated that NO possessed inhibitory effects on SARS-CoV replication. Moreover, growing evidence has shown that inhaled NO can reduce inflammatory cell-mediated lung injury by inhibiting neutrophil activation and subsequent pro-inflammatory cytokine release. Due to its potent and selective pulmonary vasodilation, inhaled NO can lower pulmonary vascular resistance and decrease edema in the alveolar spaces, which enhances ventilation/perfusion matching. In addition, recent evidence also suggests that inhaled NO could have a wide range of systemic effects via cGMP-dependent and -independent mechanisms leading to a decrease in vascular tone, and a reduced risk of thrombosis and leukocyte adhesion to pulmonary and systemic vascular endothelium [5]. Because NO acts as a pro-inflammatory and an anti-inflammatory agent, depending on the amount of NO generation and its source, early and timely initiation of inhaled NO therapy may prevent cytokine storms following abnormal vascular endothelium/leukocyte interactions.

In severe COVID-19 ARDS with hypoxemia despite optimizing ventilation and other rescue strategies, extracorporeal membrane oxygenation (ECMO) is the final therapeutic option [1]. However, given the high running cost, limited number of devices, and the skilled medical staff required to perform ECMO, inhaled NO, which is relatively of low cost and readily available, may be a promising interventional therapy for patients with severe COVID-19 ARDS. Because inhaled NO has been extensively applied to treat pulmonary hypertension, ARDS, and other respiratory diseases with a relatively good safety profile, we advocate for a clinical trial exploring the use of inhaled NO for the management of COVID-19 ARDS to be conducted as a matter of urgency.

## Data Availability

Not applicable.

## Abbreviations

COVID-19: Coronavirus disease 2019
ARDS: Acute respiratory distress syndrome
NO: Nitric oxide
SARS-CoV: Severe acute respiratory syndrome coronavirus
ECMO: Extracorporeal membrane oxygenation
